# RETRACTION: Assessing Postoperative Wound Infection Rates in Ultrasound‐Guided Microwave Ablation Versus Conventional Surgery for Varicose Veins

**DOI:** 10.1111/iwj.70431

**Published:** 2025-03-26

## Abstract

**RETRACTION:** WuJ. , LuW. , ChengG. , HuQ. , JiangB. , and LiaoS. , “Assessing Postoperative Wound Infection Rates in Ultrasound‐Guided Microwave Ablation Versus Conventional Surgery for Varicose Veins,” International Wound Journal 21, no. 4 (2024): e14584, 10.1111/iwj.14584.38112035 PMC10961856

The above article, published online on 19 December 2023, in Wiley Online Library (http://onlinelibrary.wiley.com/), has been retracted by agreement between the journal Editor in Chief, Professor Keith Harding; and John Wiley & Sons Ltd. Following an investigation by the publisher, all parties have concluded that this article was accepted solely on the basis of a compromised peer review process. The editors have therefore decided to retract the article. The authors did not respond to our notice regarding the retraction.

**RETRACTION:** J. Wu , W. Lu , G. Cheng , Q. Hu , B. Jiang , and S. Liao , “Assessing Postoperative Wound Infection Rates in Ultrasound‐Guided Microwave Ablation Versus Conventional Surgery for Varicose Veins,” International Wound Journal 21, no. 4 (2024): e14584, 10.1111/iwj.14584.38112035 PMC10961856

The above article, published online on 19 December 2023, in Wiley Online Library (http://onlinelibrary.wiley.com/), has been retracted by agreement between the journal Editor in Chief, Professor Keith Harding; and John Wiley & Sons Ltd. Following an investigation by the publisher, all parties have concluded that this article was accepted solely on the basis of a compromised peer review process. The editors have therefore decided to retract the article. The authors did not respond to our notice regarding the retraction.

